# False Recognition in Behavioral Variant Frontotemporal Dementia and Alzheimer's Disease—Disinhibition or Amnesia?

**DOI:** 10.3389/fnagi.2016.00177

**Published:** 2016-07-20

**Authors:** Emma C. Flanagan, Stephanie Wong, Aparna Dutt, Sicong Tu, Maxime Bertoux, Muireann Irish, Olivier Piguet, Sulakshana Rao, John R. Hodges, Amitabha Ghosh, Michael Hornberger

**Affiliations:** ^1^Neuroscience Research AustraliaSydney, NSW, Australia; ^2^Department of Clinical Neurosciences, University of CambridgeCambridge, UK; ^3^Norwich Medical School, University of East AngliaNorwich, UK; ^4^Australian Research Council Centre of Excellence in Cognition and its DisordersSydney, NSW, Australia; ^5^Department of Neurology and Cognitive Neurology Unit, Apollo Gleneagles HospitalKolkata, India; ^6^School of Psychology, University of New South WalesSydney, NSW, Australia; ^7^School of Medical Sciences, University of New South WalesSydney, NSW, Australia

**Keywords:** frontotemporal dementia, Alzheimer's disease, memory, recognition, disinhibition

## Abstract

Episodic memory recall processes in Alzheimer's disease (AD) and behavioral variant frontotemporal dementia (bvFTD) can be similarly impaired, whereas recognition performance is more variable. A potential reason for this variability could be false-positive errors made on recognition trials and whether these errors are due to amnesia *per se* or a general over-endorsement of recognition items regardless of memory. The current study addressed this issue by analysing recognition performance on the Rey Auditory Verbal Learning Test (RAVLT) in 39 bvFTD, 77 AD and 61 control participants from two centers (India, Australia), as well as disinhibition assessed using the Hayling test. Whereas both AD and bvFTD patients were comparably impaired on delayed recall, bvFTD patients showed intact recognition performance in terms of the number of correct hits. However, both patient groups endorsed significantly more false-positives than controls, and bvFTD and AD patients scored equally poorly on a sensitivity index (correct hits—false-positives). Furthermore, measures of disinhibition were significantly associated with false positives in both groups, with a stronger relationship with false-positives in bvFTD. Voxel-based morphometry analyses revealed similar neural correlates of false positive endorsement across bvFTD and AD, with both patient groups showing involvement of prefrontal and Papez circuitry regions, such as medial temporal and thalamic regions, and a DTI analysis detected an emerging but non-significant trend between false positives and decreased fornix integrity in bvFTD only. These findings suggest that false-positive errors on recognition tests relate to similar mechanisms in bvFTD and AD, reflecting deficits in episodic memory processes and disinhibition. These findings highlight that current memory tests are not sufficient to accurately distinguish between bvFTD and AD patients.

## Introduction

Alzheimer's disease (AD) and behavioral variant frontotemporal dementia (bvFTD) are the two most common early-onset dementias (Ratnavalli et al., 2002). Revised diagnostic criteria for both have been proposed, highlighting dysexecutive and neuropsychiatric symptoms as hallmark features in bvFTD, whereas a major feature of AD is pronounced impairment in episodic memory (McKhann et al., 1984; Rascovsky et al., 2007). However, recent studies have found significant memory deficits in bvFTD patients, including those with pathological confirmation and those in the early disease stages (Hornberger et al., 2010; Irish et al., 2011, 2014; Pennington et al., 2011). Conversely, AD patients occasionally present with executive dysfunction and “frontal” behavioral features (McKhann et al., 1984; Binetti et al., 1996). Thus, differentiating between bvFTD and AD can be challenging considering these clinical overlaps (Mendez et al., 1993; Varma et al., 1999).

Episodic memory processes have long been attributed to the integrity of the hippocampus and supporting medial temporal lobe (MTL) regions (Milner, 1970; Squire et al., 2004). Hippocampal atrophy in particular is a hallmark feature of early AD (Braak and Braak, 1991), and is considered to underpin the prominent memory deficits in these patients (Leon et al., 1996; Jack et al., 1997). Hippocampal changes have also been found in early-stage bvFTD in terms of *post-mortem* pathology (Broe et al., 2003; Kril and Halliday, 2004) and along with supporting MTL structures in *in vivo* imaging studies (Seeley et al., 2008; Whitwell et al., 2009; Hornberger et al., 2012), with the severity of atrophy in these regions sometimes comparable to that found in AD (van de Pol et al., 2006; Rabinovici et al., 2008; Pennington et al., 2011). Beyond these MTL structures, a larger neural network known as the Papez circuit has since been implicated in memory processing. This circuit, in addition to the hippocampus, consists of the fornix, mammillary bodies, anterior thalamus, and cingulate cortex. Indeed, several of these regions have been found to be compromised in both AD and bvFTD (Hornberger et al., 2012; Irish et al., 2014), specifically the anterior cingulate and thalamus in bvFTD, and posterior hippocampus and cingulate cortex in AD. Furthermore, diffusion tensor imaging (DTI) has been used to show the degeneration of the fornix in both AD (Copenhaver et al., 2006; Mielke et al., 2009) and more recently in bvFTD (Hornberger et al., 2012). The fornix is of particular relevance as it links the MTL to ventromedial prefrontal areas via the anterior commissure (Jay et al., 1992). Indeed, the episodic memory deficits in bvFTD have also been linked to the prominent prefrontal cortex (PFC) changes seen in these patients (Simons et al., 2002b; Pennington et al., 2011), which is suggested to relate specifically to deficits in the organization and implementation of memory retrieval strategies rather than true amnesia or forgetting of encoded material (Kramer et al., 2005). Memory deficits have also been associated with PFC regions in AD (Wong et al., 2014), but this prefrontal involvement has been suggested to be to a lesser extent than in bvFTD (Pennington et al., 2011). Two recent studies, however, indicate that bvFTD show impaired episodic memory even after controlling for encoding performances and semantic cueing of items to recall to compensate for PFC related memory deficits (Bertoux et al., 2014; Lemos et al., 2014). As such, it remains unclear as to how these structural changes in the PFC and Papez memory circuit regions are related to the neuropsychological profile of these patients.

On a behavioral level, AD patients exhibit accelerated forgetting, which typically manifests as poor performance on standard tests of free recall of previously learned material following a time delay (de Toledo-Morrel et al., 2000). Recently, bvFTD patients have also been found to show comparable deficits on such measures of delayed free recall (Hornberger et al., 2010; Ranjith et al., 2010; Pennington et al., 2011; Frisch et al., 2013; Irish et al., 2014). Evidence therefore indicates that recall performance is impaired in both diseases and that it appears to rely on the integrity of both MTL and PFC structures (Frisch et al., 2013; Irish et al., 2014). A different picture emerges for performance on cued recall, or recognition trials, for which bvFTD patients tend to perform better than AD patients (Hornberger et al., 2010), and sometimes show no impairment compared with healthy controls (Pasquier et al., 2001; Harciarek and Jodzio, 2005; Hutchinson and Mathias, 2007; Ricci et al., 2012). This difference that emerges between bvFTD and AD on recognition compared to free recall is attributed to the cue enabling bvFTD patients to overcome retrieval problems, and that the deficits in AD reflect a forgetting of items. This relative improvement on recognition measures between bvFTD and AD, however, appears variable, with some reports of equal impairment between these groups (Ranjith et al., 2010; Pennington et al., 2011; Irish et al., 2014). A possible reason for this inconsistency could be that both bvFTD and AD patients commit many false positive errors on recognition trials (Pasquier et al., 2001; Ricci et al., 2012), such that they incorrectly endorse many distractor items that were not presented during the encoding phase of the test. Therefore, the standard outcome measure of correct hits, or the number of target items subjects correctly identified on recognition trials, is unlikely to capture the underlying cause of memory deficits in these patients. Indeed, the over-endorsement of distractor items during recognition tasks could be either due to genuine amnesia or disinhibited responding, or a combination of both. Exploring this distinction between genuine amnesia and disinhibited behavior in recognition memory in bvFTD and AD may reveal critical differences between the recognition profiles in the two diseases.

The current study directly addresses this issue by investigating recognition performance on the Rey Auditory Verbal Learning Test (RAVLT) in bvFTD compared to AD in a large, multi-center sample. Importantly, to overcome the problem of false positive errors obscuring true recognition performance, we introduced a sensitivity index to account for false positives by subtracting these errors from the total number of correct hits, using a method derived from recent similar papers investigating recognition accuracy (Ricci et al., 2012). This approach was chosen to increase the accuracy of true correct hit detection by removing potential “noise” created from over-endorsement of distractor items. In addition, we investigated gray and white matter atrophy correlates of recognition false positive errors to elucidate whether different prefrontal and memory circuit regions were responsible for these recognition memory performance patterns. Finally, data were collected on the Hayling test (Burgess and Shallice, 1997), a verbal test of inhibitory function, to determine whether these false positive errors could be related to deficits in inhibitory processes. Based on previous findings, we expected that both bvFTD and AD patients would be impaired on delayed recall measures. Similarly, we expected that both patient groups would endorse a large number of false positives during the recognition trials. We hypothesized, however, that recognition impairment would reflect divergent cognitive mechanisms in each patient group, with deficits in AD reflecting a pure amnesia in contrast with a more disinhibited response style in the bvFTD group. As such, we predicted that discrete gray and white matter neural substrates would be associated with these deficits in each patient group, with bvFTD patients showing significantly greater PFC involvement, and AD patients showing greater involvement of Papez circuit regions.

## Materials and methods

### Case selection

Retrospective data from a total of 116 patients were used, consisting of 39 bvFTD and 77 AD patients. Patient data were sourced from the FRONTIER Dementia Clinic, Sydney, Australia (29 bvFTD, 37 AD), and the Department of Neurology and Cognitive Neurology Unit, Apollo Gleneagles Hospital, Kolkata, India (10 bvFTD, 40 AD). All bvFTD patients met current consensus FTD diagnostic criteria, had imaging confirmation of their diagnosis, and met criteria for probable bvFTD (Rascovsky et al., 2011). We included bvFTD patients with memory deficits if they fulfilled the other diagnostic criteria. Patients with no evidence of disease progression and brain atrophy on structural MRI were excluded to avoid potential non-progressive phenocopy cases. All AD patients met NINCDS-ADRDA diagnostic criteria (McKhann et al., 2011), with atypical cases excluded when they presented with significantly predominant visual or language deficits rather than memory impairment. A sample of 61 healthy volunteers, including spouses and carers of patients tested in Sydney (44) and India (18) served as control participants. High-resolution T1 MR coronal images were available for all participants from Sydney (*n* = 66). Demographic, neuropsychological, and imaging data were taken from the first clinical presentation of the patients.

The South Eastern Sydney Local Health District and the University of New South Wales ethics committees, and the local ethics committee for Apollo Gleneagles Hospitals approved the study. Participants or their person responsible provided written informed consent in accordance with the Declaration of Helsinki.

### Neuropsychological assessment

Severity of overall cognitive impairment was measured in patients and controls using the Mini-Mental State Examination (MMSE) (Folstein et al., 1983): a brief assessment of arithmetic, memory, and orientation functioning scored out of a possible 30 points. Verbal learning and memory were assessed using the Rey Auditory Verbal Learning Test (RAVLT) (Schmidt, 1996) in English at both centers. The RAVLT consists of five learning trials of 15 words, an interference trial of 15 words, followed by immediate and 30-min delayed recall, and a 50 word recognition trial including all 15 words from the learning trials, all 15 words from the interference trial, and 20 word semantically or phonologically related to these two lists. Outcomes analyzed include long term percent retention (LTPR), i.e., the percentage of final learning recalled following a 30-minute delay (delayed recall/learning trial 5 ^*^ 100); number of correct hits in the recognition trial; false positives, or number of words incorrectly positively identified in the recognition trial; and a recognition sensitivity index (correct hits—false positives) to account for over-endorsement on recognition.

The Hayling Test (Burgess and Shallice, 1997) assesses inhibition of a prepotent response by employing a sentence completion task. In the first part of the test, participants are required to complete a sentence with a single word that makes sense, as quickly as they can. The second part of the test requires the subject to complete sentences with single words that are unconnected to the meaning of the sentence. Response times are measured for both sections, as well as errors made in the second section. Errors are categorized as either Category A errors, which include words provided in the second section that meaningfully complete the sentence, or Category B errors, which are words that are semantically connected to words which meaningfully complete the sentence. The measure of disinhibition from the Hayling test used in this study was the total of both Category A and Category B errors. The Hayling Test data were available for Sydney patients only.

### Neuroimaging

#### Image acquisition and voxel-based morphometry (VBM) analysis

All Sydney patients and controls underwent the same imaging protocol with whole-brain T1-weighted images using a 3T Phillips MRI scanner with a standard quadrature head coil (8 channels). Imaging data were not available for analysis from the Indian sample. The 3D T1-weighted sequences were acquired as follows: coronal orientation, matrix 256 × 256, 200 slices, 1 × 1 mm^2^ in-plane resolution, slice thickness 1 mm, TE/TR = 2.6/5.8ms, flip angle 8 degree. 3D T1-weighted sequences were analyzed using FSL-VBM, a voxel-based morphometry analysis (Ashburner and Friston, 2000; Good et al., 2001), which is part of the FLS software package (http://www.fmrib.ox.ac.uk/fsl/fslvbm/index.html) (Smith et al., 2004). Following brain extraction from the images, tissue segmentation was carried out using the FMRIB Automatic Segmentation Tool (FAST) (Zhang et al., 2001). The resulting gray matter partial volume maps were aligned to the Montreal Neurological Institute standard space (MNI52) using the nonlinear registration approach with FNIRT (Andersson et al., 2007), which uses a b-spline representation of the registration warp field (Rueckert et al., 1999). To correct for local expansion or contraction, the registered partial volume maps were modulated by dividing them by the Jacobian of the warp field. The modulated images were then smoothed with an isotropic Gaussian kernel with a standard deviation of 2 mm (FWHM: 8 mm). Next, a voxel-wise general linear model (GLM) was applied and permutation-based non-parametric testing (with 5000 permutations per contrast) was used to form clusters with the Threshold Free Cluster Enhancement (TFCE) method (Smith and Nichols, 2009). A ROI mask for Papez memory circuit and prefrontal brain regions was created using the Harvard-Oxford cortical and subcortical structural atlas. The following atlas regions were included in the mask: hippocampus, parahippocampal gyrus, temporal fusiform gyrus, temporal pole, thalamus, mammillary bodies, cingulate cortex, inferior frontal gyrus and orbitofrontal cortex.

As a first step, differences in gray matter intensities between patients and controls were assessed (see Supplementary Table 1 for more details). For comparisons between patients and controls, a threshold of 50 contiguous voxels was used, with Family-wise Eror (FWE) correction at the *p* < 0.05 threshold. Next, correlations between gray matter atrophy and RAVLT false positives and Hayling AB error scores were entered as covariates in the design matrix of the VBM analysis in separate analyses for AD and bvFTD patients combined with controls. This procedure has previously been used in similar studies including bvFTD and AD patients (Irish et al., 2014) and serves to achieve greater variance in behavioral scores, thereby increasing the statistical power to detect brain-behavior relationships. Finally, an overlap analysis was conducted to identify common regions of gray matter atrophy correlating with both RAVLT false positives and Hayling total error scores. For all covariate analyses, a threshold of 50 contiguous voxels was used, uncorrected at the *p* < 0.001 threshold. Regions of significant atrophy were superimposed on the MNI standard brain, with maximum coordinates provided in MNI space. Areas of significant gray matter loss were localized with reference to the Harvard-Oxford probabilistic cortical and subcortical atlas.

#### Diffusion tensor imaging (DTI) analysis

The DTI-weighted sequences were acquired as follows: 32 gradient direction DTI sequence (repetition time/echo time/inversion time: 8400/68/90 ms; *b*-value = 1000 s/mm^2^; 55 2.5-mm horizontal slices, end resolution: 2.5 × 2.5 × 2.5 mm^3^; field of view 240 × 240 mm, 96 × 96 matrix; repeated twice). Two DTI sequences were acquired for each participant, which were in a first step then averaged and corrected for eddy current distortions, and this averaged sequence was used for analysis. Tract-based Spatial Statistics from FSL were used to perform a skeleton-based analysis of white matter fractional anisotropy. Fractional anisotropy maps of each individual subject were eddy current corrected and co-registered using non-linear registration using FNIRT to the MNI standard space using the FMRIB58_fractional anisotropy template, which is available as part of the FSL software. The template was sub-sampled at 2 × 2 × 2 mm due to the coarse resolution of native DTI data (i.e., 2.5 × 2.5 × 2.5 mm). After image registration, fractional anisotropy maps were averaged to produce a group mean fractional anisotropy image. A skeletonization algorithm was applied to the group mean fractional anisotropy image to define a group template of the lines of maximum fractional anisotropy, assumed to correspond to centers of white matter tracts. Fractional anisotropy values for each individual subject were then projected onto this group template skeleton. Clusters were tested using permutation-based non-parametric testing as described for the VBM analysis. Clusters reported have significance at *p* < 0.05, corrected for multiple comparisons across Family-Wise Error (FWE) correction space, unless otherwise stated. Similar to the VBM analysis, a mask for the regions of interest (fornix) was created based on the probabilistic JHU White-Matter Tractography Atlas (Mori et al., 2005). The DTI analysis first compared fornix integrity between bvFTD and controls, AD and controls, and between patient groups. Fornix integrity was then correlated with the false positive errors from the RAVLT recognition trial.

#### Statistical analysis

Clinical data were analyzed using IBM SPSS 20.0 (SPSS Inc., Chicago, IL). Preliminary analysis of distribution normality using Kolmogorov-Smirnov tests revealed deviations from normal distributions for all variables of interest, and as a result non-parametric Kruskal-Wallis, Mann-Whitney and Chi-square tests for group comparisons as well as Spearman's rank-order coefficient were used for between-group comparisons and correlations where appropriate. Effect size for the comparison between bvFTD and AD for the correct hits score on recognition was also calculated using Cohen's *d*, and percentage overlap between the two groups was derived from this.

## Results

### Demographics

Groups did not differ significantly in number of years of age or education, and patient groups did not differ in disease duration (*p*'s < 0.05). Chi-square analysis also showed no differences in sex distributions between groups, (*p*>0.05). Groups significantly differed on MMSE, with *post-hoc* comparisons revealing that both bvFTD and AD patients performed worse than controls (*p*'s < 0.001). No significant differences between patient groups were present (*p's* > 0.05).

### Neuropsychological assessment

Table 1 depicts demographics and cognitive test performance across all groups.

**Table 1:** **Mean (SD) scores and comparisons between groups for demographics and cognitive tests**.

	**bvFTD**	**AD**	**Control**	**Overall**	**bvFTD vs. AD**	**bvFTD vs. control**	**AD vs. control**
*N*	39	77	61				
Age	60.56 (7.54)	64.17 (8.25)	63.59 (6.54)	n.s.	–	–	–
Education (years)	12.28 (2.89)	12.65 (3.08)	13.14 (3.29)	n.s.	–	–	–
Sex (M/F)	26/13	42/35	30/31	n.s.	–	–	–
Disease duration (months)	41.38 (27.73)	33.06 (27.15)	–	n.s.	–	–	–
MMSE (/30)	24.18 (5.12)	21.96 (5.84)	29.13 (1.1)	^***^	n.s.	^***^	^***^
LTPR (%)	52.34 (55.01)	19.80 (27.83)	85.36 (20.50)	^***^	^**^	^***^	^***^
**RECOGNITION**							
Correct hits	11.89 (3.60)	10.44 (2.99)	13.60 (1.53)	^***^	^**^	n.s.	^***^
False positives	12.47 (11.05)	12.60 (7.13)	1.89 (2.24)	^***^	n.s.	^***^	^***^
Sensitivity index	−0.58 (10.23)	−2.16 (7.01)	11.71 (3.00)	^***^	n.s.	^***^	^***^
Hayling errors	37.36 (27.82)	16.92 (17.70)	2.10 (3.53)	^***^	n.s.	^***^	^***^

^*^indicates significant differences between groups using Mann-Whitney post-hoc tests; MMSE, Mini-Mental State Examination; LTPR, Long Term Percent Retention; Sensitivity Index, Recognition correct hits minus false positives; ^*^p < 0.05;

*** p < 0.01;

*** p < 0.001; n.s., non-significant.

Comparisons across study centers (Sydney vs. Kolkata) revealed no significant difference for any demographic (age, sex distribution, education, disease duration) or RAVLT variables (*p's* > 0.05), however the MMSE was significant lower for both bvFTD (*p* < 0.01) and AD patient groups in Kolkata (*p* < 0.001) (Supplementary Table 2).

### RAVLT

Overall group comparisons revealed significant differences on all RAVLT measures (*p*'s < 0.001), as shown in Table 1. *Post-hoc* comparisons showed that both patient groups performed worse than controls on all RAVLT measures (*p*'s < 0.001) with the exception of recognition, where bvFTD patients did not differ from controls (*p* > 0.05). AD patients performed worse than bvFTD patients on LTPR, and recognition, (*p*'s < 0.05). When false positives were taken into account using the recognition sensitivity index, both patient groups showed equally impaired performance compared to controls (*p*'s < 0.001). Notably, however, patient groups did not differ on the recognition sensitivity index or number of false positives (*p*'s = 0.06 and 0.07, respectively). The same pattern of significance emerged for all RAVLT outcomes when considering the Sydney sub-set alone (Supplementary Table 3). An effect size calculation for the comparison between bvFTD and AD across both centres for the correct hits score on recognition produced a Cohen's *d* of 0.4, which corresponds to 72.6% overlap between the two groups.

### The hayling test

In comparison to controls, both bvFTD and AD patients made significantly more total errors on the Hayling test (*p*'s < 0.001), with no significant difference between patient groups (*p*>0.07) (Table 1). Interestingly, the Hayling total errors correlated with RAVLT-recognition false-positives in both bvFTD (*R* = 0.65, *p* < 0.001) and AD, (*R* = 0.42, *p* = 0.012) but not in controls (*R* = −0.06, *p* = 0.71).

### Neuroimaging

#### VBMNeural correlates of RAVLT-recognition false positives

**Figure 1:**
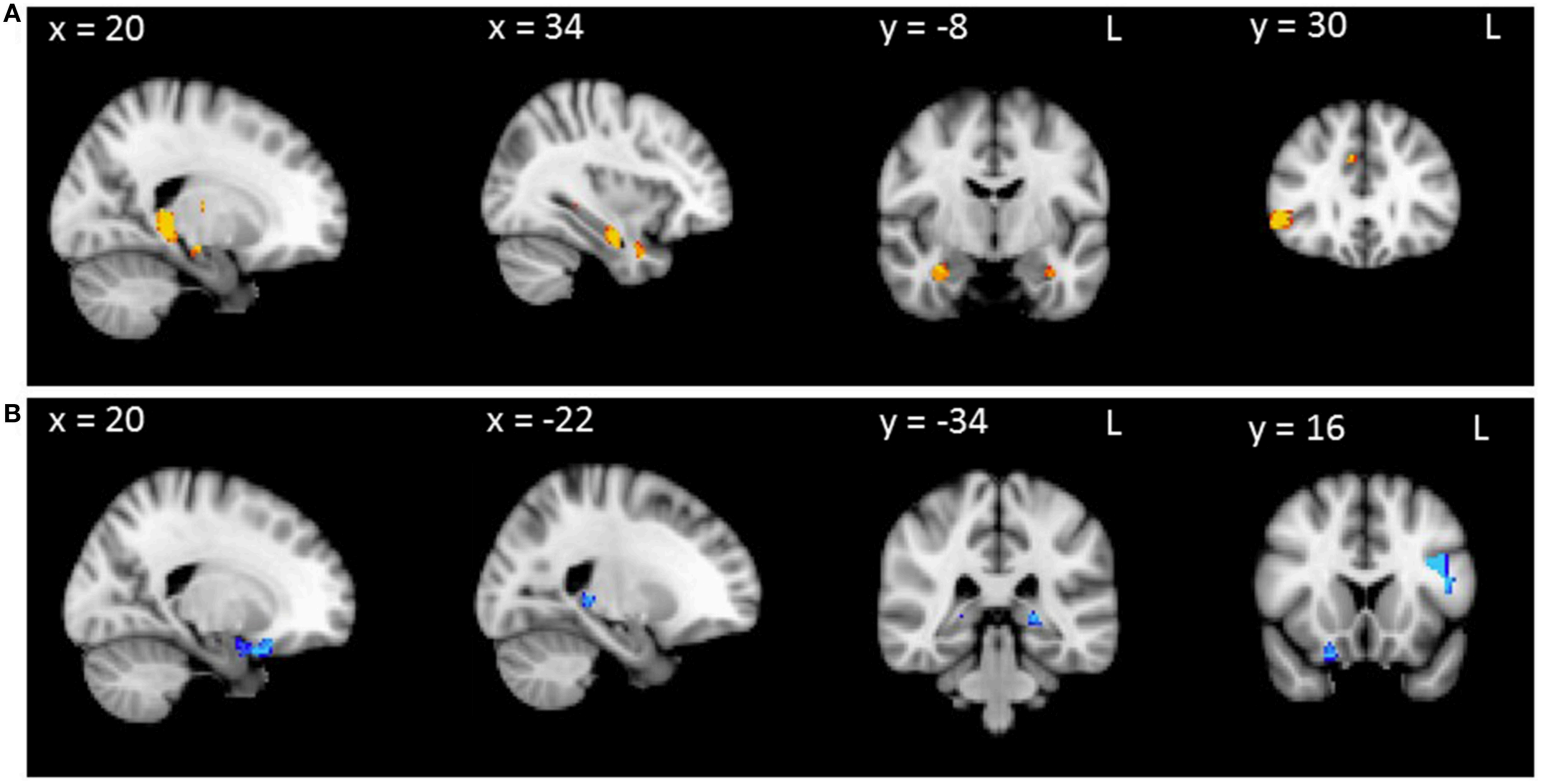
**VBM analyses showing brain regions in which gray matter intensity correlates significantly with RAVLT false positives in (A) AD patients compared with controls and (B) bvFTD patients compared with controls**. Colored voxels show regions that were significant in the analysis with *p* < 0.001 uncorrected, with a cluster threshold of 50 contiguous voxels. Clusters are overlaid on the MNI standard brain.

In AD patients combined with controls, false positives covaried with regions of gray matter density in the right temporal pole, orbitofrontal cortex and parahippocampal gyrus, as well as left inferior frontal gyrus, hippocampus and thalamus (Table 2, Figure 1A). False positives in bvFTD patients combined with controls correlated with regions of gray matter density in the parahippocampal gyrus bilaterally, as well as the right hippocampus, thalamus, temporal pole, orbitofrontal cortex and inferior frontal gyrus (Table 2, Figure 1B).

**Table 2:** **VBM analyses showing brain regions in which gray matter intensity correlates significantly with RAVLT false positives in patient groups combined with controls**.

**Regions**	**Hemisphere (L/R)**	**MNI Coordinates**			**Number of voxels**
		***X***	***Y***	***Z***	
**bvFTD COMBINED WITH CONTROLS**					
Parahippocampal gyrus (anterior), hippocampus,	R	34	−10	−26	277
Hippocampus, thalamus	R	22	−32	−8	264
Parahippocampal gyrus (anterior)	L	−12	−16	−28	160
Orbitofrontal cortex, inferior frontal gyrus	R	46	28	−6	134
Temporal pole	R	34	6	−32	97
**AD COMBINED WITH CONTROLS**					
Temporal pole, orbitofrontal cortex, parahippocampal gyrus (anterior)	R	24	6	−24	184
Inferior frontal gyrus	L	−44	12	12	98
Hippocampus, thalamus	L	−28	−40	0	64

All results uncorrected at p < 0.001; only clusters with at least 50 contiguous voxels included. All clusters reported t > 3.90. MNI, Montreal Neurological Institute.

#### Neural correlates of disinhibition

**Figure 2:**
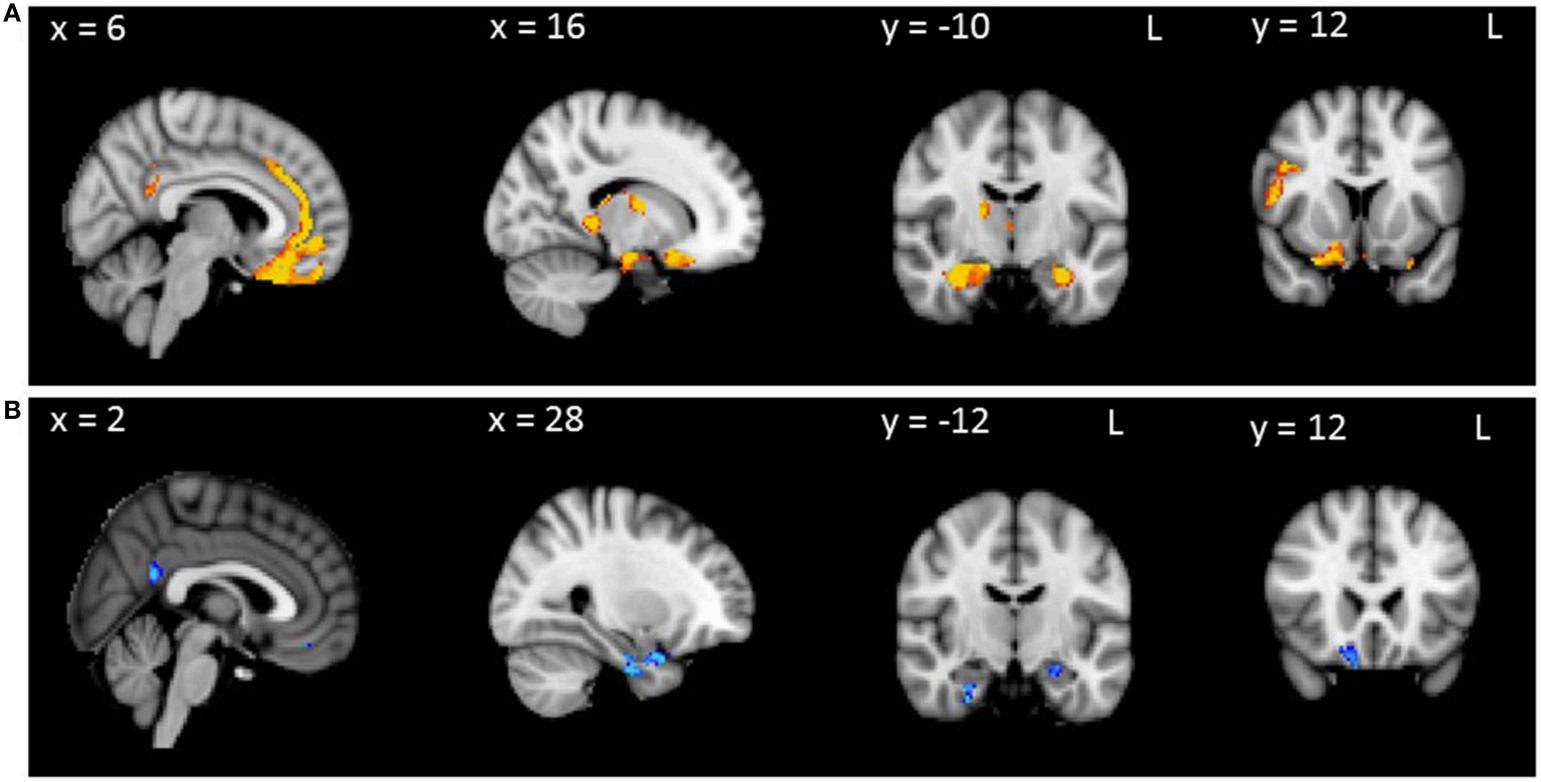
**VBM analyses showing brain regions in which gray matter intensity correlates significantly with Hayling total errors in (A) AD patients compared with controls and (B) bvFTD patients compared with controls**. Colored voxels show regions that were significant in the analysis with *p* < 0.001 uncorrected, with a cluster threshold of 50 contiguous voxels. Clusters are overlaid on the MNI standard brain.

To investigate neural correlates of disinhibition in AD and bvFTD, Hayling total error scores were entered as covariates in the design matrix of separate VBM analyses. In AD patients combined with controls, Hayling total error scores correlated with atrophy in the right temporal pole, orbitofrontal cortex, parahippocampal gyrus, precuneus, posterior cingulate cortex and hippocampus (Table 3, Figure 2A). In contrast, Hayling total error scores in bvFTD patients combined with controls covaried with regions of atrophy in the bilateral parahippocampal gyrus, and hippocampus, left fusiform cortex, as well as right temporal pole, orbitofrontal cortex, subcallosal cortex, medial prefrontal cortex, paracingulate cortex, anterior and posterior cingulate cortices, inferior frontal gyrus and thalamus (Table 3, Figure 2B).

**Table 3:** **VBM analyses showing brain regions in which gray matter intensity correlates significantly with Hayling total error scores in patient groups combined with controls**.

**Regions**	**Hemisphere (L/R)**	**MNI Coordinates**			**Number of voxels**
		***X***	***Y***	***Z***	
**bvFTD COMBINED WITH CONTROLS**					
Temporal pole, parahippocampal gyrus (anterior), orbitofrontal cortex, subcallosal cortex, medial prefrontal cortex paracingulate cortex, anterior cingulate cortex, inferior frontal gyrus,	R	34	6	−32	3676
Fusiform cortex (anterior), parahippocampal gyrus (anterior), hippocampus	L	−36	−6	−32	493
Inferior frontal gyrus	R	52	10	8	147
Parahippocampal gyrus (posterior), hippocampus	L	−14	−34	−8	113
Thalamus	R	18	−10	10	75
Posterior cingulate cortex	R	6	−50	22	69
**AD COMBINED WITH CONTROLS**					
Temporal pole, parahippocampal gyrus (anterior), orbitofrontal cortex	R	26	6	−28	322
Precuneus, posterior cingulate cortex	R	6	−52	18	101
Parahippocampal gyrus (anterior), hippocampus	R	28	−12	−34	65

All results uncorrected at p < 0.001; only clusters with at least 50 contiguous voxels included. All clusters reported t > 3.90. MNI, Montreal Neurological Institute.

#### Overlap analysis

**Figure 3:**
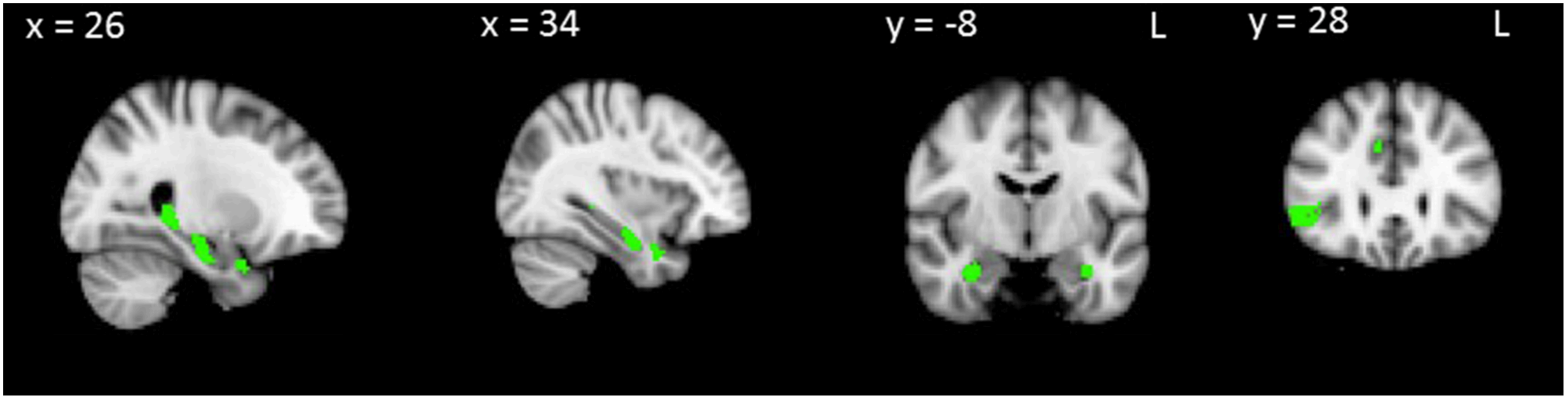
**VBM analyses showing brain regions in which gray matter intensity correlates significantly with both RAVLT false positives and Hayling total errors in bvFTD combined with controls**. Colored voxels show regions that were significant in the analysis with *p* < 0.001 uncorrected, with a cluster threshold of 50 contiguous voxels. Clusters are overlaid on the MNI standard brain.

Next, we conducted an overlap analysis to investigate common regions underlying false positives and disinhibition in AD and bvFTD (Table 4). In bvFTD combined with controls, common regions for false positives and Hayling total error scores were identified in the hippocampus, orbitofrontal cortex and inferior frontal gyrus (Table 4, Figure 3). No regions significantly overlapped in the AD group combined with controls.

**Table 4:** **VBM results showing common regions of significant gray matter intensity decrease that correlate with RAVLT false positives and Hayling total errors in bvFTD combined with controls**.

**Regions**	**Hemisphere (L/R/B)**	**MNI Coordinates**			**Number of voxels**
		***X***	***Y***	***Z***	
**FALSE POSITIVES AND HAYLING TOTAL ERROR SCORES**					
Hippocampus	R	22	−32	−8	222
Hippocampus	R	34	−10	−26	186
Orbitofrontal cortex, inferior frontal gyrus	R	46	28	−6	114

All results uncorrected at p < 0.001; only clusters with at least 50 contiguous voxels included. All clusters reported t > 3.90. MNI, Montreal Neurological Institute.

#### DTI

In the DTI analysis, a contrast between healthy controls and patient groups revealed better fornix integrity in the control group. A direct comparison of fornix integrity between patient groups revealed that the bvFTD group showed significantly more fornix degeneration than the AD group.

There was a trend between false-positive errors and decreased fornix integrity in the bvFTD group (not combined with controls) (*r* = 0.39, *p* = 0.06), however this was not statistically significant. No other groups showed a significant or emerging relationship between false positive errors and fornix integrity.

## Discussion

The aim of this study was to compare recognition profiles in bvFTD and AD, with a view to establishing the cognitive and neural mechanisms differentially contributing to recognition impairments in these syndromes. Our findings indicate significant overlap between these groups both clinically and in terms of neural mechanisms, with suggestion of more involvement of disinhibition and its associated neural regions in bvFTD, and a slightly more amnestic profile in AD. More specifically, despite the finding that bvFTD patients performed at a similar level to controls in terms of correct hits on the recognition test, the overlap between bvFTD and AD (72.6%) prevents any accurate clinical distinction. Importantly, both bvFTD and AD patients showed similarly high false recognition rates in comparison to controls. Furthermore, the imaging analysis revealed considerably overlapping regions of atrophy that correlate with these false positives in bvFTD and AD, with both groups showing the conjunct involvement of Papez circuit and prefrontal regions.

In more detail, despite both bvFTD and AD patients showing impairment on delayed recall, which is in line with past investigations comparing these groups (Hornberger et al., 2010; Ranjith et al., 2010; Pennington et al., 2011; Irish et al., 2014), only AD patients showed significant deficits on the standard recognition measure of correct hits, as well as showing greater impairment on delayed recall. However, both AD and bvFTD patients endorsed significantly more false positive errors compared to healthy controls, which is a recurrent finding (Pasquier et al., 2001; Ricci et al., 2012). Although the standard recognition outcome of correct hits was significantly higher in bvFTD compared to AD, this distinction has poor clinical significance due to the large overlap (72.6%) and was no longer significant once false positives were accounted for using the sensitivity index. These findings contribute to a growing body of evidence which indicates that bvFTD patients can indeed show considerable deficits across a range of memory measures (Hornberger et al., 2010; Ranjith et al., 2010; Pennington et al., 2011; Irish et al., 2014). The difference between the standard recognition measure and the sensitivity index also highlights the limited value of standard memory outcomes in explaining true memory performance in these patients. Specifically, although bvFTD patients showed a normal performance on correct hits, they were equally impaired as AD patients once false positive errors were considered. When considered together, it appears that bvFTD patients in particular endorse many extra words in addition to those correctly identified.

Regarding the imaging analysis, there was considerable overlap in neural correlates of false positive errors between bvFTD and AD. Both groups showed involvement of Papez circuit regions, particularly medial temporal and thalamic structures, as well as PFC regions. This overlap corroborates similar findings from studies relating neural regions to memory recall performance (Irish et al., 2014). Contrary to the Irish et al. study (Irish et al., 2014) however, there was less of a distinction between anterior and posterior network involvement in bvFTD and AD, respectively—although the current study used a ROI approach compared to the whole brain analysis conducted by Irish and colleagues. However, another possible reason for this is that the false positive errors would be underpinned more strongly by source memory deficits and selecting the correct information to retrieve, which in addition to being linked to frontal atrophy (Simons et al., 2002a), particularly the OFC (Collette and Van der Linden, 2002), has also been associated with MTL regions (Söderlund et al., 2008). However, it should also be noted that the AD patients in this sample were age-matched to the bvFTD group, such that many of the AD patients would be considered to belong to the early-onset form of AD. It has previously been shown that, in comparison to later-onset AD, these patients can have a pattern of neural atophy which is less restricted to the characteristic MTL regions. Overall, these findings suggest a reciprocal relationship between prefrontal and Papez circuit regions, particularly between the MTL and PFC, reflecting possible deficits in both memory retention and retrieval. Taken together with the behavioral data, these retrieval deficits could specifically reflect poor inhibition of incorrect responses on recognition, particularly in the bvFTD group.

We explored this hypothesis further by investigating the relationship between false positive errors and disinhibition errors on the Hayling test, a validated test of verbal inhibition. These two outcomes correlated in both patient groups, but when taking the correlation coefficient as a measure of effect size, the link between false positive errors and desinhibition was stronger in bvFTD. However, it should also be noted that bvFTD patients were slightly more impaired on the Hayling, although this difference did not reach significance, nonetheless this could have contributed to the strength of its relationship with false positive errors between groups. On a neuroanatomical level, our imaging findings fit with previous studies, showing ventromedial prefrontal and anterior temporal involvement linked to the disinhibition errors in bvFTD (Hornberger et al., 2011); however, the current study also showed MTL and thalamic involvement in both patient groups for this measure. Taken together, this strongly suggests that poor inhibition of retrieving or endorsing incorrect items made on recognition was related to poor memory performance to a greater extent in bvFTD, in addition to memory deficits. However, this was also present, albeit to a lesser degree, in AD patients, highlighting PFC involvement in false recognition suppression in this group in line with suggestions from previous findings (Budson et al., 2002). Taken together, these findings suggest a considerable overlap in mechanisms underlying the false positive errors in bvFTD and AD, with a slight double dissociation of greater disinhibition in bvFTD and memory deficits in AD.

This study involved a large sample size, with behavioral data from multiple studies, that allowed us to reach good statistical power for the behavioral analysis of the recognition performance in bvFTD and AD, as compared to previous quantitative investigations of memory performance between bvFTD and AD. Delineation of correct hits and false positive errors on the recognition trial using the sensitivity index allowed us to explore specific memory impairment in both diseases. Finally, we used a multimodal imaging analysis (VBM and DTI) to explore the differential gray and white matter correlates of false positive recognition and disinhibition. Despite these strengths, a potential limitation of this study that pathological data were not available for the bvFTD patients and as such, it remains unclear whether any of this sample had underlying AD pathology. Furthermore, there were no Hayling test or imaging data available for the Indian sample, so several of the critical analyses could only be conducted on the Sydney sample. However, participants were matched between both centers for all other variables, including RAVLT scores, with MMSE as the only exception. The use of data from two centers also calls into question the consistency between sites in terms of diagnostic accuracy; however diagnoses were made in expert centers with a multi-disciplinary approach and according to the same consensus criteria (McKhann et al., 2011; Rascovsky et al., 2011). Also, the clinical progression over a minimum 12 months follow-up was in accordance of the initial diagnosis for all patients. Finally, our results did not allow for us to investigate structured vs. unstructured recall, which would be a worthwhile direction for future investigations. Future investigations would also benefit from incorporating post-mortem confirmation of pathology or *in vivo* investigation of AD pathology using techniques such as amyloid PET imaging or CSF biomarkers.

These findings have clinical significance. The behavioral results from this study corroborate findings from a growing body of evidence suggesting that performance on standard memory tests does not adequately distinguish bvFTD from AD (Hornberger et al., 2010; Ranjith et al., 2010; Pennington et al., 2011; Irish et al., 2014). It particularly calls into question the inclusion of “relative sparing of episodic memory” as a diagnostic criterion for bvFTD (Rascovsky et al., 2011), considering that we and others (Hornberger et al., 2010, 2012; Ranjith et al., 2010; Pennington et al., 2011; Irish et al., 2014) have demonstrated that memory deficits in these patients share some similarities with those observed in AD, in terms of severity and underlying neural processes. Novel findings from this study call into question the suitability of standard memory measures, particularly recognition, to adequately reflect memory deficits and differentiate between bvFTD and AD. The correct diagnosis of these diseases is important in terms of differing treatment and management implications. As such, the reliance on memory performance in the clinic to distinguish these groups may not be sufficient. There is emerging evidence that other domains such as spatial orientation (Tu et al., 2015) and social cognition (Bertoux et al., 2015) are very reliable for distinguishing bvFTD from AD, and these tests can be very easily administered in a clinical setting. Our findings also have theoretical implications regarding our understanding of the prefrontal involvement in memory dysfunction, particularly in bvFTD, such that the characteristic disinhibited profile of these patients might also contribute to poor performance on memory measures in addition to an amnesia similar to AD. As such, development of tests that can disentangle such contributions to memory impairment in bvFTD and AD is recommended, in order to improve upon existing memory measures and better distinguish between these patient groups.

## Author contributions

EF and MH contributed to the study design. Statistical analysis were performed by EF, SW, and ST. EF, SW, AD, SR, MI, OP, JH, AG contributed to the data acquisition. EF, SW, MB, MH were involved in the interpretation of findings. EF and MH contributed to the paper writing. All authors were involved in the final manuscript revision.

### Conflict of interest statement

The authors declare that the research was conducted in the absence of any commercial or financial relationships that could be construed as a potential conflict of interest.
