# Supplementary material for: False Recognition in Behavioral Variant Frontotemporal Dementia and Alzheimer's Disease—Disinhibition or Amnesia?
Source: Front Aging Neurosci. 2016 Jul 20;8:177. doi: 10.3389/fnagi.2016.00177 (PMC4951525; doi:10.3389/fnagi.2016.00177)
Supplement: Supplementary file 1 [file Table1.DOCX]

*Supplementary Table 1.* VBM results showing regions of significant grey matter intensity decrease for AD and bvFTD groups compared to controls. All results FWE corrected at *p*<.05; only clusters with at least 100 contiguous voxels included. All clusters reported *t*>2.51. MNI = Montreal Neurological Institute.

| **Regions** | **Hemisphere (L/R/B)** | **MNI Coordinates** | | | **Number of voxels** |
| --- | --- | --- | --- | --- | --- |
|  |  | **X** | **Y** | **Z** |  |
| **AD < controls** |  |  |  |  |  |
| Parahippocampal gyrus, hippocampus, temporal pole, temporal fusiform cortex, thalamus, posterior cingulate cortex, medial prefrontal cortex, orbitofrontal cortex, subcallosal cortex | B | 20 | 2 | -26 | 3778 |
| Inferior frontal gyrus | L | -36 | 14 | 26 | 1511 |
| Hippocampus, parahippocampal gyrus, temporal fusiform cortex, temporal pole, thalamus, orbitofrontal cortex | L | -24 | -12 | -18 | 1504 |
| Posterior cingulate cortex | B | -2 | -54 | 26 | 185 |
| **bvFTD < controls** |  |  |  |  |  |
| Temporal fusiform cortex, hippocampus, parahippocampal cortex, temporal pole, orbitofrontal cortex, medial prefrontal cortex, anterior cingulate cortex, subcallosal cortex, inferior frontal gyrus, thalamus | B | -24 | -8 | -52 | 14366 |
| Inferior frontal gyrus | L | -34 | 16 | 26 | 156 |
| **bvFTD < AD** |  |  |  |  |  |
| Temporal fusiform cortex, parahippocampal gyrus, hippocampus, amygdala, thalamus, temporal pole, orbitofrontal cortex, medial prefrontal cortex, subcallosal cortex, inferior frontal gyrus, anterior cingulate gyrus, paracingulate gyrus  **AD < bvFTD**  None | B | -24 | -12 | -44 | 9127 |
