# Supplementary material for: False Recognition in Behavioral Variant Frontotemporal Dementia and Alzheimer's Disease—Disinhibition or Amnesia?
Source: Front Aging Neurosci. 2016 Jul 20;8:177. doi: 10.3389/fnagi.2016.00177 (PMC4951525; doi:10.3389/fnagi.2016.00177)
Supplement: Supplementary file 2 [file Table2.DOCX]

*Supplementary Table 2:* Mean (SD) Scores and Comparisons of Demographics and Test Outcomes across India and Sydney Patient Groups.

|  | bvFTD | | | AD | | |
| --- | --- | --- | --- | --- | --- | --- |
|  | India | Sydney |  | India | Sydney |  |
| *n* | 10 | 29 |  | 40 | 37 |  |
| Age | 60.60 (9.17) | 60.55 (7.09) | n.s. | 64.20 (8.99) | 64.14 (7.49) | n.s. |
| Sex (M/F) | 7/3 | 19/10 | n.s. | 21/19 | 21/16 | n.s. |
| Education (Y) | 13.50 (2.46) | 11.85 (2.94) | n.s. | 13.00 (3.21) | 12.34 (2.97) | n.s. |
| Disease Duration (Y) | 38.00 (30.38) | 42.55 (27.23) | n.s. | 28.78 (21.70) | 37.09 (31.23) | n.s. |
| MMSE (/30) | 19.20 (6.48) | 25.90 (3.19) | ** | 19.97 (6.84) | 24.00 (3.67) | *** |
| RAVLT |  |  |  |  |  |  |
| LTPR (%) | 33.16 (38.29) | 58.95 (58.81) | n.s. | 15.33 (23.88) | 24.63 (31.17) | n.s. |
| Correct Hits | 11.56 (2.74) | 12.00 (3.88) | n.s. | 10.35 (3.08) | 10.54 (2.93) | n.s. |
| False Positives | 13.89 (8.27) | 12.00 (11.94) | n.s. | 13.60 (6.71) | 11.51 (7.50) | n.s. |
| Sensitvity Index | -2.33 (9.07) | 0.00 (10.69) | n.s. | -3.25 (6.16) | -0.97 (7.74) | n.s. |

*Note. ** indicates significant differences between groups using Mann Whitney tests; MMSE = Mini-Mental State Examination; LTPR = Long Term Percent Retention; Sensitivity Index = correct hits minus false positives; *p<.05; **p<.01; ***p<.001; n.s.= non-significant.
