# Supplementary material for: False Recognition in Behavioral Variant Frontotemporal Dementia and Alzheimer's Disease—Disinhibition or Amnesia?
Source: Front Aging Neurosci. 2016 Jul 20;8:177. doi: 10.3389/fnagi.2016.00177 (PMC4951525; doi:10.3389/fnagi.2016.00177)
Supplement: Supplementary file 3 [file Table3.DOCX]

*Supplementary Table 3:* Mean (SD) Scores and Comparisons Between Groups for Demographics and Cognitive Tests in Sydney Only.

|  | bvFTD | AD | Control | Overall | bvFTD vs. AD | bvFTD vs. Control | AD vs. Control |
| --- | --- | --- | --- | --- | --- | --- | --- |
| *N* | 29 | 37 | 43 |  |  |  |  |
| Age | 60.55 (7.08) | 64.14 (7.49) | 64.58 (5.04) | * | - | * | - |
| Education (Years) | 11.85 (2.94) | 12.34 (2.97) | 13.28 (2.61) | n.s. | - | - | - |
| Sex (M/F) | 19/10 | 21/16 | 19/24 | n.s. | - | - | - |
| Disease duration (Months) | 42.55 (27.23) | 37.09 (31.23) | - | n.s. | - | - | - |
| MMSE (/30) | 25.90 (3.19) | 24.0 (3.67) | 29.33 (.84) | *** | n.s. | *** | *** |
| LTPR (%) | 58.95 (58.81) | 24.63 (31.17) | 85.22 (20.92) | *** | * | ** | *** |
| Recognition |  |  |  |  |  |  |  |
| Correct Hits | 12.0 (3.88) | 10.54 (2.93) | 13.61 (1.37) | *** | ** | n.s. | *** |
| False Positives | 12.0 (11.94) | 11.47 (7.50) | 2.0 (2.35) | *** | n.s. | *** | *** |
| Sensitivity Index | 0 (10.69) | -.97 (7.74) | 11.61 (3.13) | *** | n.s. | *** | *** |
| Hayling Errors | 38.92 (28.40) | 16.92 (17.70) | 2.22 (3.60) | *** | n.s. | *** | *** |

*Note. ** indicates significant differences between groups using Mann-Whitney *post hoc* tests; MMSE = Mini-Mental State Examination; LTPR = Long Term Percent Retention; Sensitivity Index = Recognition correct hits minus false positives; *p<.05; **p<.01; ***p<.001; n.s.= non-significant.
